# Low CD4/CD8 ratio is associated with increased morbidity and mortality in late and non-late presenters: results from a multicentre cohort study, 2004–2018

**DOI:** 10.1186/s12879-022-07352-z

**Published:** 2022-04-15

**Authors:** Lourdes Domínguez-Domínguez, Marta Rava, Otilia Bisbal, Luis Lopez-Cortés, Joaquín Portilla, Daniel Podzamczer, Julián Olalla, Daniel Fuster, Rafael Rubio, Inmaculada Jarrín, José Antonio Iribarren, Santiago Moreno, Cohort of the Spanish HIV/AIDS Research Network (CoRIS)

**Affiliations:** 1 Unidad Infección VIH, Hospital Universitario 12 de Octubre, Departamento de Medicina, Facultad de Medicina, Universidad Complutense de Madrid, Instituto de Investigación Sanitaria Hospital 12 de Octubre (imas12), Madrid, Spain; 2 Centro Nacional de Epidemiología, Instituto de Salud Carlos III, Avda. Monforte de Lemos 5, 28029 Madrid, Spain; 3 CIBER de Enfermedades Infecciosas (CIBERInfec), Madrid, Spain; 4 Enfermedades Infecciosas, Microbiología Clínica y Medicina Preventiva, Instituto de Biomedicina de Sevilla/Hospital Universitario Virgen del Rocío/CSIC/Universidad de Sevilla, Seville, Spain; 5 Hospital General Universitario de Alicante/Fundación para el Fomento de la Investigación Sanitaria y Biomédica de la Comunitat Valenciana/Departamento de Medicina Clínica, Universidad Miguel Hernández, Alicante, Spain; 6 Unidad VIH, Servicio de Enfermedades Infecciosas, IDIBELL-Hospital Universitari de Bellvitge, Barcelona, Spain; 7 Unidad de Medicina Interna, Hospital Costa del Sol, Marbella, Spain; 8 Servicio de Medicina Interna, Unidad de Adicciones, Hospital Universitari Germans Trias i Pujol, Universitat Autònoma de Barcelona, Redes de Investigación Cooperativa Orientadas al Resultado en Salud (RICORS) RD21/0009/0004, Badalona, Spain; 9 Hospital Universitario de Donostia, IIS Biodonostia, San Sebastián, Spain; 10 Servicio de Enfermedades Infecciosas, Hospital Ramón y Cajal, IRYCIS: Universidad de Alcalá, Madrid, Spain

**Keywords:** Late presentation, CD4/CD8, Clinical outcomes, AIDS events, Serious non-AIDS events, Mortality

## Abstract

**Background:**

To study whether the association between the CD4/CD8 ratio variation over time and the development of clinical outcomes vary in late presenters (CD4 count < 350/µL or AIDS event at enrolment) or advanced presenters (CD4 count < 200/µL or AIDS event at enrolment).

**Methods:**

We included ART-naïve adults from the Cohort of the Spanish HIV/AIDS Research Network (CoRIS) enrolled between January 2004 up to November 2018 and with at least 6 months of follow-up. We used extended Cox proportional hazard models to estimate the hazard ratios (HRs) for the association between CD4/CD8 ratio over time and a composite endpoint of the occurrence of the first AIDS event, first serious non-AIDS event or overall mortality occurring from 6 months after enrolment. HRs in non-late, late and advanced presenters were obtained by including an interaction term between late presentation status and CD4/CD8 ratio over time.

**Results:**

Of 10,018 participants, 55.6% were late presenters and 26.5% were advanced presenters. Compared with CD4/CD8 ratio > 0.4, CD4/CD8 ratio ≤ 0.4 over time was associated with an increased risk of experiencing the composite endpoint in non-late (HR 1.90; 95%CI 1.48, 2.43), late (HR 1.94; 1.46, 2.57) and advanced presenters (HR 1.72; 1.26, 2.34). Similarly, CD4/CD8 ratio ≤ 0.4 over time was associated with a higher risk of developing an AIDS event (HR 3.31; 2.23, 4.93 in non-late; HR 2.75; 1.78, 4.27 in late and HR 2.25; 1.34, 3.76 in advanced presenters) or serious non-AIDS event (HR 1.39; 0.96, 2.02 in non-late, HR 1.62; 1.10, 2.40 in late and HR 1.49; 0.97, 2.29 in advanced presenters) as well as with a higher risk of overall mortality (HR 1.49; 0.92, 2.41 in non-late, HR 1.80; 1.04, 3.11 in late and HR 1.61; 0.92, 2.83 in advanced presenters) compared to CD4/CD8 > 0.4, regardless of the late presentation status.

**Conclusions:**

A low CD4/CD8 measured over time is associated with increased risk of morbidity and mortality in people living with HIV independently of their late presentation status. These data support the prognostic role of CD4/CD8 over time and can help defining a subgroup of patients who need closer monitoring to avoid comorbidities.

**Supplementary Information:**

The online version contains supplementary material available at 10.1186/s12879-022-07352-z.

## Background

Mortality in people living with HIV (PLWH) has decreased since the advent of highly active antiretroviral treatment (ART) [1]. Nonetheless comorbidities are increasing [2, 3] and they are particularly high in those who present late for diagnosis [4, 5].

In this scenario, efforts have been made to find prognostic markers of clinical events [6]. The CD4+ T cells/CD8+ T cells ratio (CD4/CD8 ratio) has shown an association with both AIDS and non-AIDS events in virologically suppressed individuals [7]. Particularly, a CD4/CD8 ratio ≤ 0.4 has been found to be associated with T cell activation, innate immune activation and an immunosenescent phenotype, and to predict serious non-AIDS events [8, 9], even in virologically suppressed persons with absolute CD4 count > 500/µL.

Although mortality and morbidity are especially high in late presenters [4, 10] the potential prognostic role of the CD4/CD8 ratio has not been studied yet in this population. Our aim is to evaluate whether the variation in CD4/CD8 ratio over time is associated with the development of AIDS and non-AIDS events and mortality in participants from the cohort of the Spanish HIV/AIDS Research Network (CoRIS) and to study whether these associations vary with late presentation and advanced disease.

## Methods

### Study design

CoRIS is an open, prospective multicentre cohort of persons with confirmed HIV infection, naïve to ART at study entry, recruited in 46 centres from 13 Autonomous Regions in Spain from 2004-onwards, as described in detail in [11]. CoRIS Study Groups are listed in Additional file 1.

### Study population

We included CoRIS participants recruited from January 2004 to November 2018, age ≥ 18 years, with available information on late presentation at enrolment, at least 6 months of follow up, at least one determination of CD4 count and CD8 count from the 6th month after enrolment, and information on the clinical outcomes of the composite endpoint. Individuals who were monitored in the three centres not providing data on non-AIDS events were excluded.

### Definitions of late presentation

Late presenters were participants with CD4 count below 350/µL and/or with an AIDS event occurred before the 24th week after enrolment regardless of the CD4 cell count, both conditions met before ART initiation. Advanced presenters were participants with CD4 count below 200/µL and/or with an AIDS event occurred before the 24th week after enrolment regardless of the CD4 cell count, both conditions met before ART initiation [12]. By definition, all  advanced presenters were also late presenters. We considered as non-late presenters participants with CD4 count greater than 350/µL and no AIDS events.

### Health outcomes

The primary endpoint was clinical progression, defined as a composite endpoint of the occurrence of the first AIDS or serious non-AIDS event or death from any cause occurred from 6 months after enrolment. As serious non-AIDS event we considered any of the following conditions: cardiovascular events (myocardial infarction, angina, heart disease, transient ischemic attack, reversible ischemic deficit, stroke and peripheral arteriopathy or death from cardiovascular disease), renal events (end-stage renal disease, initiation of dialysis or renal transplantation, or death from renal disease), liver events (ascites, digestive haemorrhage due to oesophageal varices, hepatic encephalopathy, liver transplantation, or death from liver disease), non-AIDS malignancy or death from non-AIDS malignancy, and infectious-related deaths.

### Statistical methods

We classified the participants as having low (≤ 0.4) or high (> 0.4) CD4/CD8 ratio: the cut-off at 0.4 correspond to the first quartile of the overtime distribution of CD4/CD8 ratio and was chosen based on its association with serious non-AIDS events [8]. Since both the CD4 and the CD8 count and their ratio vary over time and its variation may affect the risk of developing a clinical outcome, we modelled the CD4/CD8 ratio as a time-varying covariate. We estimated the hazard ratios (HRs) and 95% confidence intervals (CI) for the association with the clinical outcomes with extended Cox proportional hazard models [13].

Briefly, the follow-up period for each person was broken up into subintervals, corresponding to the CD4 and CD8 measurements. The time-varying covariates values and the endpoint were updated at each subinterval up to the occurrence of the first event or the censoring time. We performed two sets of analysis: one set including non-late and late presenters and another one including non-late and advanced presenters. To evaluate whether the association between the CD4/CD8 ratio and the clinical outcomes varied with the late presentation status, we included an interaction term between late presentation and over time CD4/CD8 ratio in the multivariable Cox models, which allowed us to obtain HRs for the association of interest in non-late presenters, late presenters and advanced presenters.

Multivariable models were adjusted for age (< 50, ≥ 50 years) and CD4 count (< 500, ≥ 500/µL), that were both modelled as time-varying covariates, a combined variable of gender and HIV transmission category (men who have sex with men, injection drug use, heterosexual women, heterosexual men, other/unknown), educational level (no education or primary education alone, secondary education, university, other/unknown), region of origin (Europe, Sub-Saharan Africa, Latin America, other/unknown) and presence of hepatitis C virus antibodies (no, yes or unknown), presence of hepatitis B surface antigen (no, yes or unknown) and viral load (< 10,000, 10,000–100,000, ≥ 100,000 copies/mL, unknown) at enrolment.

All statistical analyses were performed using R version 4.0 [14] and extended Cox regression models were estimated with the survival package [13, 15].

### Sensitivity analyses

To account for the role of viral suppression (viral load < 50 copies mL) in the association between the CD4/CD8 ratio and the clinical outcomes, we included the viral load as a time-varying variable (< 50, ≥ 50 copies/mL). Further, in order to evaluate whether early ART initiation modify the associations under study, we repeated the analyses after stratification for timing of ART initiation (< 1 year vs ≥ 1 year after the enrolment).

## Results

During the study period, 10,018 participants met the inclusion criteria. Out of them, 5574 (55.6%) were non-late presenters, 4444 (44.4%) were late presenters and 2659 (26.5%) were advanced presenters (Table 1).

**Table 1:** Characteristics at enrolment of non-late presenters, late presenters and advanced presenters

	All participants, n = 10,018	Non-late presenters, n = 5574, 55.6%	Late presenters, n = 4444, 44.4%	Advanced presenters, n = 2659, 26.5%
Age (years)				
Median (1st, 3rd quartile)	35.5 (29.1 43.1)	33. 4 (27.7 40.4)	38.6 (31.4 46.1)	40.2 (33.4 47.6)
< 30	2469 (24.6%)	1696 (30.4%)	773 (17.4%)	327 (12.3%)
30–49	6290 (62.8%)	3422 (61.4%)	2868 (64.5%)	1742 (65.5%)
≥ 50	1259 (12.6%)	456 (8.2%)	803 (18.1%)	590 (22.2%)
Transmission group (n (%))				
MSM	6071 (60.6%)	3910 (70.1%)	2161 (48.6%)	1120 (42.1%)
IDU	707 (7.1%)	280 (5.0%)	427 (9.6%)	295 (11.1%)
Heterosexual women	1334 (13.3%)	616 (11.1%)	718 (16.2%)	451 (17.0%)
Heterosexual men	1560 (15.6%)	633 (11.4%)	927 (20.9%)	645 (24.3%)
Other/unknown	346 (3.5%)	135 (2.4%)	211 (4.7%)	148 (5.6%)
Educational level (n (%))				
Primary education or less	1390 (13.9%)	574 (10.3%)	816 (18.4%)	546 (20.5%)
Secondary education	4598 (45.9%)	2648 (47.5%)	1950 (43.9%)	1185 (44.6%)
University	2395 (23.9%)	1548 (27.8%)	847 (19.1%)	421 (15.8%)
Other/unknown	1635 (16.3%)	804 (14.4%)	831 (18.7%)	507 (19.1%)
Region of origin (n (%))				
Europe	7498 (74.8%)	4307 (77.3%)	3191 (71.8%)	1918 (72.1%)
Sub-Saharan Africa	439 (4.4%)	169 (3.0%)	270 (6.1%)	173 (6.5%)
Latin America	1875 (18.7%)	992 (17.8%)	883 (19.9%)	495 (18.6%)
Other/unknown	206 (2.1%)	106 (1.9%)	100 (2.3%)	73 (2.7%)
CD4 count, cells/µL				
Median (1st; 3rd quartile)	400 (215 600)	566 (450 739)	189 (75 275)	93 (38 162)
< 200	2293 (22.9%)	0 (0.0%)	2293 (51.6%)	2293 (86.2%)
200–350	1943 (19.4%)	0 (0.0%)	1943 (43.7%)	158 (5.9%)
350–500	2061 (20.6%)	2007 (36.0%)	54 (1.2%)	54 (2.0%)
≥ 500	3614 (36.1%)	3567 (64.0%)	47 (1.1%)	47 (1.8%)
Unknown	107 (1.1%)	0 (0.0%)	107 (2.4%)	107 (4.0%)
Nadir CD4 count, cells/µL				
Median (1st; 3rd quartile),	351 (192 520)	499 (407 644)	179 (72 263)	89 (36 157)
< 200	2235 (22.3%)	29 (0.5%)	2206 (49.6%)	2100 (79.0%)
200–500	4016 (40.1%)	2309 (41.4%)	1707 (38.4%)	179 (6.7%)
≥ 500	2369 (23.6%)	2335 (41.9%)	34 (0.8%)	34 (1.3%)
Unknown	1398 (14.0%)	901 (16.2%)	497 (11.2%)	346 (13.0%)
CD4/CD8 ratio, Median (1st; 3rd quartile)	0.42 (0.22, 0.66)	0.58 (0.41, 0.81)	0.21 (0.11, 0.36)	0.14 (0.07, 0.23)
≤ 0.4	3841 (38.3%)	1107 (19.9%)	2734 (61.5%)	1798 (67.6%)
> 0.4	4225 (42.2%)	3529 (63.3%)	696 (15.7%)	184 (6.9%)
Unknown	1952 (19.5%)	938 (16.8%)	1014 (22.8%)	677 (25.5%)
Viral load (n (%)), copies/mL				
< 10,000	1627 (16.2%)	1252 (22.5%)	375 (8.4%)	149 (5.6%)
10,000–100,000	3751 (37.4%)	2315 (41.5%)	1436 (32.3%)	652 (24.5%)
> 100,000	3135 (31.3%)	1076 (19.3%)	2059 (46.3%)	1460 (54.9%)
Unknown	1505 (15.0%)	931 (16.7%)	574 (12.9%)	398 (15.0%)
Aids diagnosis (n (%))	1339 (13.4%)	0 (0.0%)	1339 (30.1%)	1339 (50.4%)
Hepatitis C virus antibodies (n (%))				
No	8021 (80.1%)	4615 (82.8%)	3406 (76.6%)	1982 (74.5%)
Yes	975 (9.7%)	410 (7.4%)	565 (12.7%)	373 (14.0%)
Unknown	1022 (10.2%)	549 (9.8%)	473 (10.6%)	304 (11.4%)
Hepatitis B virus surface antigen (n (%))				
No	7719 (77.1%)	4117 (73.9%)	3602 (81.1%)	2129 (80.1%)
Yes	325 (3.2%)	158 (2.8%)	167 (3.8%)	112 (4.2%)
Unknown	1974 (19.7%)	1299 (23.3%)	675 (15.2%)	418 (15.7%)

All p-values for the comparisons between late and non-late presenters were < 0.001. All p-values for the comparisons between advanced and non-late presenters were < 0.001

*MSM* men who have sex with men, *IDU* injection drug user

Late presenters and advanced presenters were more likely than non-late presenters to have acquired HIV infection through heterosexual contact or injections, to have a lower level of education, to be from sub-Saharan Africa or Latin America, and to have enrolled at an older age and with a higher viral load (Table 1).

### Dynamic of CD4/CD8 ratio by late presentation status

**Fig. 1:**
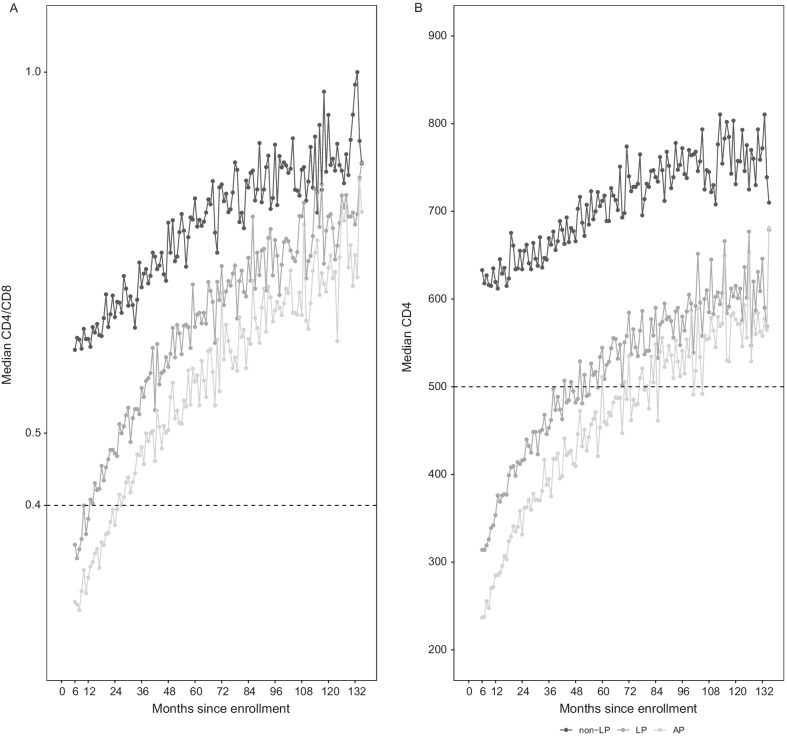
Median CD4/CD8 (**A**) and CD4 count (**B**) over time according to late presentation status. *non-LP* non-late presenters, *LP* late presenters; *AP* advanced presenters

Median CD4/CD8 ratio and CD4 count increased over time in the three groups. While non-late presenters had a median CD4/CD8 ratio above the threshold of 0.4 from 6 months after enrolment, late presenters reached it after 12 months and advanced presenters after 24 months (Fig. 1A, B).

**Fig. 2:**
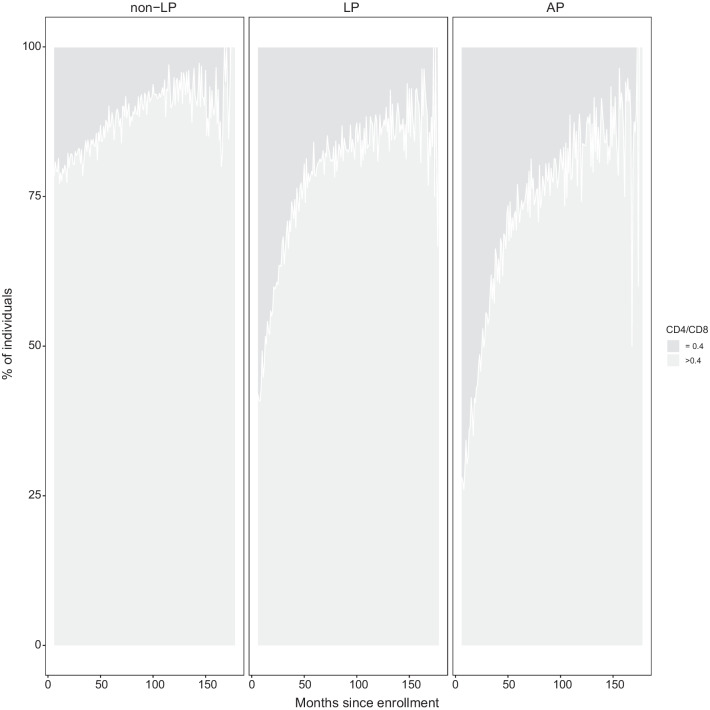
Percentage of individuals with CD4/CD8 ratio below or above 0.4 over time in non-late, late and advanced presenters. *non-LP* non-late presenters, *LP* late presenters; *AP* advanced presenters

The percentage of individuals with CD4/CD8 ≤ 0.4 was lower in non-late presenters than in late and advanced presenters during the entire follow-up. More than half of the individuals with late presentation or advanced disease had CD4/CD8 ratio ≤ 0.4 at enrolment and over time, with the highest proportion observed in advanced presenters (Fig. 2).

### Impact of dynamic of CD4/CD8 ratio on clinical outcomes (overall and by late presentation status)

**Fig. 3:**
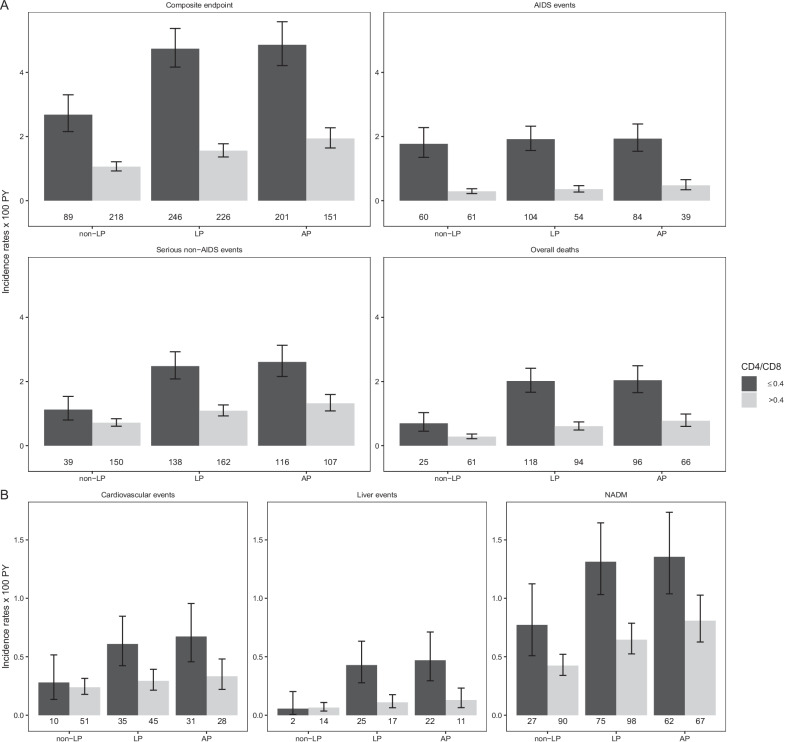
Incidence rates of the clinical outcomes of interest according to CD4/CD8 ratio over time in in non-late, late and advanced presenters. The figures below each bar correspond to the number of events. *non-LP* non-late presenters, *LP* late presenters; *AP* advanced presenters; *NADM* non-AIDS defining malignancies

Regardless of their late presentation status at enrolment, low CD4/CD8 ratio over time was associated with an increased incidence of the composite endpoint and of each of its components (Fig. 3).

As shown in Table 2, compared with CD4/CD8 ratio > 0.4, CD4/CD8 ratio ≤ 0.4 was associated with an increased risk of clinical progression in non-late (HR 1.90; 95%CI 1.48, 2.43), late (HR 1.94; 95%CI 1.46, 2.57), and advanced presenters (HR 1.72; 95%CI 1.26, 2.34). In general, after adjustment for a wide range of covariates that included the CD4 count measured over time, the adjusted HRs were attenuated compared with the unadjusted estimates.

**Table 2:** Association between CD4/CD8 ratio over time and clinical outcomes in non-late, late and advanced presenters

		Non-late presenters, CD4/CD8 ≤ 0.4 vs CD4/CD8 > 0.4	p-value	Late presenters, CD4/CD8 ≤ 0.4 vs CD4/CD8 > 0.4	p-value	Interaction p-value LP vs non-LP	Advanced presenters, CD4/CD8 ≤ 0.4 vs CD4/CD8 > 0.4	p-value	Interaction p-value AP vs non-LP
Composite endpoint	Unadjusted HR (95%CI)	2.57 (1.97, 3.35)	< 0.001	3.11 (2.33, 4.17)	< 0.001		2.55 (1.88, 3.45)	< 0.001	
	Adjusted HR (95%CI)	1.90 (1.48, 2.43)	< 0.001	1.94 (1.46, 2.57)	< 0.001	0.869	1.72 (1.26, 2.34)	< 0.001	0.598
AIDS events	Unadjusted HR (95%CI)	5.80 (3.82, 8.81)	< 0.001	4.63 (2.93, 7.33)	< 0.001		3.39 (1.99, 5.77)	< 0.001	
	Adjusted HR (95%CI)	3.31 (2.23, 4.93)	< 0.001	2.75 (1.78, 4.27)	< 0.001	0.365	2.25 (1.34, 3.76)	0.002	0.139
Serious Non-AIDS events	Unadjusted HR(95%CI)	1.62 (1.16, 2.28)	0.005	2.45 (1.69, 3.55)	< 0.001		2.12 (1.41, 3.20)	< 0.001	
	Adjusted HR (95%CI)	1.39 (0.96, 2.02)	0.079	1.62 (1.10, 2.40)	0.016	0.423	1.49 (0.97, 2.29)	0.069	0.640
Cardiovascular events	Unadjusted HR(95%CI)	1.25 (0.57, 2.73)	0.570	2.44 (0.94, 6.35)	0.067		2.50 (0.88, 7.14)	0.087	
	Adjusted HR(95%CI)	1.33 (0.64, 2.76)	0.444	1.82 (0.72, 4.61)	0.208	0.511	1.85 (0.67, 5.12)	0.234	0.425
Liver events	Unadjusted HR (95%CI)	0.84 (0.22, 3.18)	0.792	3.54 (0.81, 15.51)	0.093		3.36 (0.65, 17.44)	0.149	
	Adjusted HR (95%CI)	0.42 (0.12, 1.50)	0.183	1.54 (0.37, 6.34)	0.565	0.059	1.60 (0.33, 7.71)	0.556	0.078
Neoplasms	Unadjusted HR (95%CI)	1.92 (1.34, 2.75)	< 0.001	2.27 (1.52, 3.40)	< 0.001		1.90 (1.25, 2.88)	0.003	
	Adjusted HR (95%CI)	1.61 (1.02, 2.56)	0.043	1.56 (0.97, 2.50)	0.066	0.867	0.64 (0.04, 11.32)	0.193	0.500
Overall mortality	Unadjusted HR (95%CI)	2.63 (1.57, 4.39)	< 0.001	3.85 (2.15, 6.91)	< 0.001		3.09 (1.69, 5.64)	< 0.001	
	Adjusted HR (95%CI)	1.49 (0.92, 2.41)	0.103	1.80 (1.04, 3.11)	0.036	0.511	1.61 (0.92, 2.83)	0.095	0.734

*CI* confidence interval; *HR* hazard ratio; *LP* late presenters; *AP* advanced presenters

^a^HR (CI 95%) were estimated with extended Cox regression models adjusted for age (< 50, ≥ 50 years) and CD4 count (< 500, ≥ 500/µL), both modelled as time-varying covariates, a combined variable of gender and HIV transmission category (MSM, IDU, heterosexual women, heterosexual men, other/unknown), educational level (no studies or primary education, secondary education, university, other/unknown), region of origin (Europe, Sub-Saharan Africa, Latin America, other/unknown), presence of hepatitis C virus antibodies (no, yes or unknown), presence of hepatitis B surface antigen (no, yes or unknown) and viral load (< 10,000, 10,000–100,000, ≥ 100,000 copies/mL, unknown) at enrolment, and with an interaction term between CD4/CD8 ratio over time and late or advanced presentation

The risk of developing an AIDS event was three times higher for individuals with CD4/CD8 ratio ≤ 0.4 compared to those with CD4/CD8 ratio > 0.4 among non-late presenters and twice as high in both late and advanced presenters. The adjusted HRs for serious non-AIDS events, comparing CD4/CD8 ratio ≤ 0.4 versus CD4/CD8 ratio > 0.4, varied from 1.39 (95%CI 0.96, 2.02) in non-late to 1.62 (95%CI 1.10, 2.40) in late, and 1.49 (95%CI 0.97, 2.29) in advanced presenters. These estimates suggested that CD4/CD8 ratio ≤ 0.4 could be associated with serious non-AIDS events. However, in non-late presenters as well as in advanced presenters, possible HRs that are highly compatible with our data, given our model, ranged from 0.96 and 0.97 (essentially no association) to 2.02 and 2.29 (a relatively strong association). Similar considerations can also be extrapolated from the results on specific serious non-AIDS events.

Mortality associated with CD4/CD8 ratio ≤ 0.4 was 49% higher in non-late, 80% higher in late and 61% higher in advanced presenters. There was no evidence that the association between CD4/CD8 ratio and the clinical outcomes differed between non-late and late presenters (all p-values for interaction > 0.5) or between non-late and advanced presenters (all p-values for interaction > 0.1).

Overall, compared with CD4/CD8 ratio > 0.4, CD4/CD8 ratio ≤ 0.4 doubled the risk of experiencing the composite endpoint (HR 2.00; 95%CI 1.63, 2.45), with a three-fold increased risk of an AIDS event (3.05; 95%CI 2.25, 4.12), and it was also associated with an increased risk of serious non-AIDS event (1.58; 95%CI 1.27, 1.97), and overall mortality (1.62; 95%CI 1.32, 1.98). CD4/CD8 ratio ≤ 0.4 was also associated with increased risk of some specific serious non-AIDS events, such as cardiovascular (1.89; 95%CI 1.19, 3.00) and non-AIDS defining malignancies (1.52; 95%CI 1.10, 2.12).

### Sensitivity analysis

The analysis including viral load as time-varying variable was consistent with the main results (data not shown). In the sensitivity analysis stratified by the timing of ART initiation, the association between low CD4/CD8 ratio with clinical outcomes was confirmed, regardless on ART initiation time. In those starting ART within the 1st year since the enrolment, the HRs for the association between the CD4/CD8 and the composite endpoint was 2.34 (95%CI 1.42, 3.85, n of events = 99) in non-late, 1.86 (95%CI 1.09, 3.17, n = 507) in late and 1.75 (95%CI 1.28, 2.40, n = 399) in advanced presenters. In participants starting ART 1 year after the enrolment or later, the corresponding HRs were 1.72 (95%CI 1.23, 2.41, n = 239) for non-late, 2.43 (95%CI 1.31, 4.52, n = 54) for late and 1.03 (95%CI 0.29, 3.64, n = 27) for advanced presenters.

## Discussion

In this observational study, we found that PLWH with a CD4/CD8 ratio ≤ 0.4 over time are at a greater risk of clinical progression regardless their late presentation status. This corroborates the role of the CD4/CD8 ratio as prognostic marker of clinical outcomes in PLWH no matter how they present at diagnosis. Although this marker has previously shown an association with the development of AIDS- and non-AIDS-related events in some cohorts of PLWH [8, 9, 16–18] to the best of our knowledge this is the first study evaluating its role among late and advanced presenters and assessing whether its clinical impact is the same among the non-late presenters.

Certainly, late presentation status was found to have a negative impact on both the absolute CD4 cell count and CD4/CD8 ratio recovery, as observed in other studies [18, 19]. Besides, we found that the risk of clinical progression, and specifically of AIDS events, was higher when the CD4/CD8 ratio was ≤ 0.4 over time for both non-late and late or advanced presenters. However, the negative impact of a CD4/CD8 ≤ 0.4 ratio was also detected among non-late presenters. It is worth highlighting that we did not focus on CD4/CD8 ratio values at enrolment or at ART initiation, but on CD4/CD8 ratio values measured over time. We also observed that the negative impact of the low CD4/CD8 ratio did not depend on ART initiation timing or on viral response, suggesting that the prognostic role of the CD4/CD8 ratio may be independent on when the treatment is initiated and on its effectiveness in terms of viral suppression.

The adjusted models suggested an increased risk of serious non-AIDS events associated with low CD4/CD8 ratio. This association was observed regardless of late presentation status, although it was of lower magnitude than the one observed for the AIDS-related events and in non-late and advanced presenters was quite imprecise. Among non-AIDS events, we observed increased risk of malignancies, cardiovascular and liver events, though with large and imprecise confidence intervals, probably due to the low number of events in each category. Our results are in discordance with those of two previous studies. Castilho et al. [20] observed no association between baseline CD4/CD8 ratio and risk of non-AIDS events in a cohort of virologically suppressed HIV-positive adults, independently of the CD4+ cell count. These results are not directly comparable with ours, as the authors did not include time-varying CD4/CD8 ratio as predictor and they estimated HRs for 0.1 increase in the CD4/CD8 ratio. Using similar methods to those in our study, Hema et al. [21] did find inconclusive estimates of the association between time-varying CD4/CD8 ratio ≥ 0.5 (vs CD4/CD8 < 0.5) and serious non-AIDS events considered overall, when controlling for time-varying CD4 count. The discrepancy with our results may depend on the different definition of non-AIDS events, while, in line with our findings, the authors observed that low CD4/CD8 ratio was associated with increased risk of non-AIDS defining cancers.

The higher risk of non-AIDS events found in our study could be explained by increased inflammation and immunoactivation, that have been already identified as potential mechanisms for both atherosclerosis [22] and malignancies [23] in PLWH. Thus, our findings bring to light the presence of those phenomena specifically in individuals with low CD4/CD8 ratio [8]. Some authors have in fact reported a lower CD4/CD8 ratio in PLWH diagnosed with a cardiovascular event when compared to those without [9, 20]. Besides, earlier studies describe an association between the inversion of the CD4/CD8 ratio and surrogate markers of vascular disease, such as the carotid intima-media thickness [24, 25] and arterial stiffness [24]. In terms of neoplasms, Serrano et al. [9] found lower CD4/CD8 ratio in participants diagnosed with non-AIDS defining malignancies in comparison with those without a cancer diagnosis. A longer follow-up of our cohort it is desirable as it will allow us to observe more events and therefore make more reliable estimates to confirm the impact of low CD4/CD8 ratio on clinical outcomes.

We acknowledge that our analysis is not adjusted for some potential confounder factors, such as smoking status. Nevertheless, other studies showed higher risk of lung cancer associated with low CD4/CD8 ratio even after accounting for smoking status [26, 27]. As further limitation, we did not consider the effect of ART regimens, which may vary according to initial CD4 count. We also could not classify as late or non-late presenters those who did not have CD4 count or AIDS events available before starting treatment, although they accounted for only 3% of the entire cohort. Finally, although cohort participants were followed for an extended period, their young age may decrease our ability to evaluate outcomes which develop only over the long-term and later in life.

## Conclusions

In conclusion, a low CD4/CD8 ratio measured over time is associated with an increased risk of morbidity and mortality in PLWH independently of their late presentation status. These preliminary findings support the prognostic role of variation of CD4/CD8 ratio over time in this highly vulnerable subpopulation and can help define the subgroup of service users who may need closer monitoring to avoid comorbidities, thus optimizing the follow-up and the use of healthcare resources.

## Supplementary Information

**Additional file 1**. Title of data: Cohort of the Spanish HIV/AIDS Research Network (CoRIS). Description of data: Centres and investigators involved in CoRIS.

## Data Availability

The datasets used and/or analysed during the current study are available from the corresponding author on reasonable request.

## Abbreviations

ART: Active antiretroviral treatment
CD4/CD8ratio: CD4+ T cells/CD8+ T cells ratio
CI: Confidence intervals
CoRIS: Cohort of the Spanish HIV/AIDS Research Network
HR: Hazard ratio
PLWH: People living with HIV
